# Comparison of Electrolyte Composition and Crystallization Patterns in Bird and Reptile Tears

**DOI:** 10.3389/fvets.2020.00574

**Published:** 2020-08-13

**Authors:** Arianne P. Oriá, Ariane de J. Lacerda, Ana Cláudia S. Raposo, Nayone L. L. C. Araújo, Ricardo Portela, Marcos A. Mendonça, Ali M. Masmali

**Affiliations:** ^1^School of Veterinary Medicine and Zootechny, Federal University of Bahia, UFBA, Salvador, Brazil; ^2^Laboratory of Immunology and Molecular Biology, Institute of Health Sciences, Federal University of Bahia, Salvador, Brazil; ^3^Cornea Research Chair, Department of Optometry, College of Applied Medical Sciences, King Saud University, Riyadh, Saudi Arabia

**Keywords:** electrolyte balance, grading scale, tear composition, tear electrolyte dosage, tear ferning test, wild animal

## Abstract

To compare tear electrolytes and tear crystallization patterns in birds and reptiles, tears were sampled by Schirmer tear test from 10 animals each of *Ara ararauna, Amazona aestiva, Tyto alba, Rupornis magnirostris, Chelonoidis carbonaria*, and *Caiman latirostris*, and 5 of *Caretta caretta*. The aliquots were pooled to assess concentrations of total protein, chloride, phosphorus, iron, sodium, potassium, calcium, and urea. For the tear ferning test, samples of each species were observed under a polarized light microscope at room temperature and humidity. Crystallization patterns were graded according Rolando and Masmali scales. There was more total protein and urea in owl and sea turtle tears, respectively, than in the other animals tested. Electrolyte balance was similar for all species, with higher sodium, chloride, and iron. In birds, Rolando-scale grades of tear crystallization patterns ranged from I to II, and from 0 to 2 using the Masmali scale; in reptiles, grades were II to IV (Rolando) and 2 to 4 (Masmali). Crystallization arrangements of some species had higher scores, as caimans and sea turtles, possibly due to different the tear composition. Marine and lacustrine species presented higher. The ionic balance of lacrimal fluids of birds and reptiles was similar to that in humans, with higher values of sodium and chloride. However, a similar tear composition did not influence the crystal morphology. Crystallization classification suggested that higher grades and types are due to the different microelements present in the tears of wild species.

## Introduction

The tear film is a complex fluid that covers the ocular surface, serving as the interface between the eye and the environment (1). The main functions of the tear film are related to its interaction with the corneal epithelium, including lubrication, nutrition, metabolite removal, protection, and stability of the ocular surface (2, 3). These functions stem from the diversity of its components, and it is characterized in vertebrates by the presence of lipids, water, proteins, and electrolytes (3, 4). However, due to species heterogeneity, there are still some gaps related to the dynamics of its components, and how they act to maintain homeostasis.

Tears are synthesized from the secretory lacrimal glands, goblet cells, and epithelial cells in the cornea and conjunctiva (5). Secreted lacrimal ions have been described in mammals, resulting from the unidirectional transport of salt and water through the acinar cell layer (6). As tears are not a direct product of the blood serum, as well as urine, some studies have attempted to find biomarkers for conditions derived from a break in the blood-lacrimal gland barrier, observed in disease status. Other components of tears, including proteins and urea, are synthesized based on requirements of the adjacent epithelium, in response to the environment or blood parameters (1, 4, 7). Therefore, studies assessing this fluid's composition as related to different environments or species-specific metabolism are important to understand the balance in each condition. However, there is a lack of knowledge about the tear composition of wild animals.

Following the study of electrolyte composition in tears, supersaturation of tear components forms crystals whose patterns, when dried, can be used for qualitative evaluation; this is called the tear ferning test (TFT), as described by Tabbara and Okumoto (8). Other studies have reported use of the TFT to determine the tear composition under healthy conditions and in patients with ocular abnormalities (9, 10), in both humans (2) and other species (11–13). In humans, results have shown that the tear's osmotic density is the main trigger modifying the crystallization patterns (14); however, nothing is known for birds and reptile tears.

The TFT has been described in mammalian species, including humans (2), dogs, horses, monkeys, and camels (11–13), and the tears have been classified using different scales, such as those of Rolando (10) and Masmali et al. (2). Differences in the crystals' arrangements, including angulations and secondary formations, make the TFT a subjective test (15). The use of two scales to classify the crystallization patterns can reduce this subjectivity.

Studies on bird and reptile ocular surface components have shown differences in the lacrimal apparatus, tear production and palpebral incursion frequency among species (16, 17); however, there are only a few qualitative descriptions of tears, mainly of their microcomponents (18). The elucidation of tear composition can help in the understanding of the homeostasis process played by this specific body fluid and in the diagnosis of ocular surface diseases. Vision is the most important sense for many animal species, and ocular diseases can change their social and feeding behaviors, as well as their ability to escape from predators. A deficient vision may contribute to an increase in the mortality of these animals. Thus, the aim of the present study was to describe the tear film electrolytes and crystallization patterns in birds and reptiles and compare them to data obtained from humans.

## Materials and Methods

### Ethical Considerations

This study was approved by the System of Authorization and Information on Biodiversity (SISBIO—authorization number 27489), and by the National Genetic Patrimony Management System (SISGEN—registration number A1F8C27), both from the Brazilian Ministry of the Environment. The study also received consent from the Ethics Committee on Animal Experimentation of the School of Veterinary Medicine and Zootechnology of UFBA (protocol number 72/2016). All procedures were conducted in accordance with the Declaration of the Association for Research in Vision and Ophthalmology, and the National Institutes of Health's Guide for the Care and Use of Laboratory Animals. Stress factors, such as intense sounds and light stimuli or physical restraints for a long time, and procedures that can induce pain, such as an excessive manipulation of the eyelids and eyelashes, were prevented and reduced with the objective to preserve animal welfare during the sampling process.

Protocols involving humans were approved by the Ethics Committee in Research of the Institute of Health Science, Federal University of Bahia (protocol number 2.388.777) and met the Brazilian legislation and ethical principles of the Helsinki Declaration. Written informed consent was obtained from all individuals.

### Species

We examined 40 birds, 10 animals each of the species: *Ara ararauna* (blue-and-yellow macaw), *Amazona aestiva* (turquoise-fronted amazon), *Tyto alba* (barn owl), *Rupornis magnirostris* (roadside hawk), and 25 reptiles: 10 *Chelonoidis carbonaria* (red-footed tortoise), 10 *Caiman latirostris* (broad-snouted caiman), and 5 *Caretta caretta* (loggerhead sea turtle), all healthy adults (clinical and hematological evaluations performed by the responsible technical staff) from a triage center of wild animals (CETAS, Salvador, Brazil), a commercial breeder (Mister Cayman®, Maceió, Brazil), and a conservation center (TAMAR Project, Mata de São João, Brazil). The study was performed as part of routine physical examinations conducted by the local veterinary staff. Therefore, a physical examination was performed before the ocular examination, and animals with indications of systemic or ophthalmic diseases were excluded.

The criteria used for species selection was the inclusion of bird and reptile species belonging to different ecological niches, and animals from which it is possible to collect tear fluid samples. Also, macaws (*Ara ararauna*), parrots (*Amazona aestiva*), and tortoises (*Chelonoidis carbonaria)* were chosen because they are animals raised as pets. As a consequence of the ethical aspects regarding wild animals and native fauna (as defined by the Brazilian Ministry of the Environment) and the availability of species found in captivity that could be clinically evaluated, the number of sampled species and animals was limited.

To validate the data obtained for the wild species, 10 human volunteers (7 women and 3 men), aged 25 to 45 years, with normal values of tear production and no ocular surface abnormalities as shown by slit-lamp biomicroscopy (Kowa Company®, Torrance, CA) were included in the study. None of these patients had a prior history of neoplasms or systemic metabolic diseases, nor did they use systemic or topical medications that affect ocular homeostasis. None of them used contact lenses.

### Sampling and Dosage of Tear Electrolytes

To acquire the samples, all animals were physically restrained, with a period of 20 min between restraint and sampling for the sea turtles and caimans to prevent sample contamination with water residue. Samples were collected between March and October 2017 in the morning (0800–1130 h), following the sequence right eye then left eye. Tears were collected using a Schirmer tear test strip (Tear-Flo™ Diagnostic Strips, Tucson, AZ), except from sea turtles where collection was via syringe due to high tear viscosity. The strip was inserted 1 mm into the lower conjunctival fornix until the wetted portion reached 30 mm. The strips were then placed in 0.5-mL microtubes (Eppendorf Protein LoBind Tubes, São Paulo, Brazil) with perforated ends, inserted in a 2.0-mL microtube (Eppendorf) [adapted from (19)]. The collection site was the same for all animals (lower conjunctival sac). The tubes were centrifuged at 25,830 g for 10 min at 4°C.

To evaluate electrolytes, tear samples were pooled for each species—as described in humans and animals for tear biochemistry evaluation (13, 19)—to obtain the required volume for processing, and kept at −20°C until testing. Commercial colorimetric kits based on biochemical and enzymatic reactions were used to quantify total protein, chloride, phosphorus, iron, sodium, potassium (Labtest®, Belo Horizonte, Brazil), and calcium (Doles®, Goiânia, Brazil), following the manufacturers' recommendations for each test. All the evaluations were performed in duplicate and the results were expressed as means.

### TFT

Following centrifugation of an individual Schirmer strip, a 5.0-μL aliquot was recovered using a micropipette, placed on a glass slide, and dried under controlled temperature and humidity. Temperature and humidity were monitored (Termo-Higrômetro Equitherm® Model Th-439, Minas Gerais, Brazil) during sample collection and processing, and kept at 21–23°C and 45–52%, respectively, for all species.

After complete drying, the slides were evaluated under a 10× magnification polarized-light microscope coupled with a camera to capture the images (Zeiss Microscope, Scope A.1/AX10 Axion Cam ICc5, Oberkochen, Germany). The ferning images were classified by three separate blinded evaluators, two of them from the Veterinary Ophthalmology Research Group at Federal University of Bahia, and one from King Saud University, all experienced in the use of the grading scales. The final grade was assigned using results that were agreed upon by at least two of the three evaluators. Tear ferning patterns were classified according to the Rolando grading scale (types I, II, III, and IV) and according to the Masmali grading scale (grades 0, 1, 2, 3, and 4). The evaluators were asked to comment on the slides of each species with respect to the characteristics found, such as thickness and size of the main and secondary branches, and the space between crystals (ferns).

### Statistical Analysis

Statistical analysis was conducted using SPSS version 22.0 software (IBM, Armonk, NY), and the level of significance was set to 5% (*P* < 0.05). Shapiro–Wilk test was used to test for TFT value data normality. Wilcoxon test was used for comparison of the same variables between eyes. The Kruskal–Wallis test was used to assess significant differences between species at the same scales. A Cohen's Kappa coefficient was used to assess the agreement between of the observers' classifications for both scales and between the observers in each scale. The coefficients showed correlations that were classified as follows: slight correlation (0.00 to 0.2); fair correlation (0.21 to 0.4); moderate correlation (0.41 to 0.6); substantial correlation (0.61 to 0.8); almost perfect correlation (0.81–1.0) (20).

## Results

**Figure 1:**
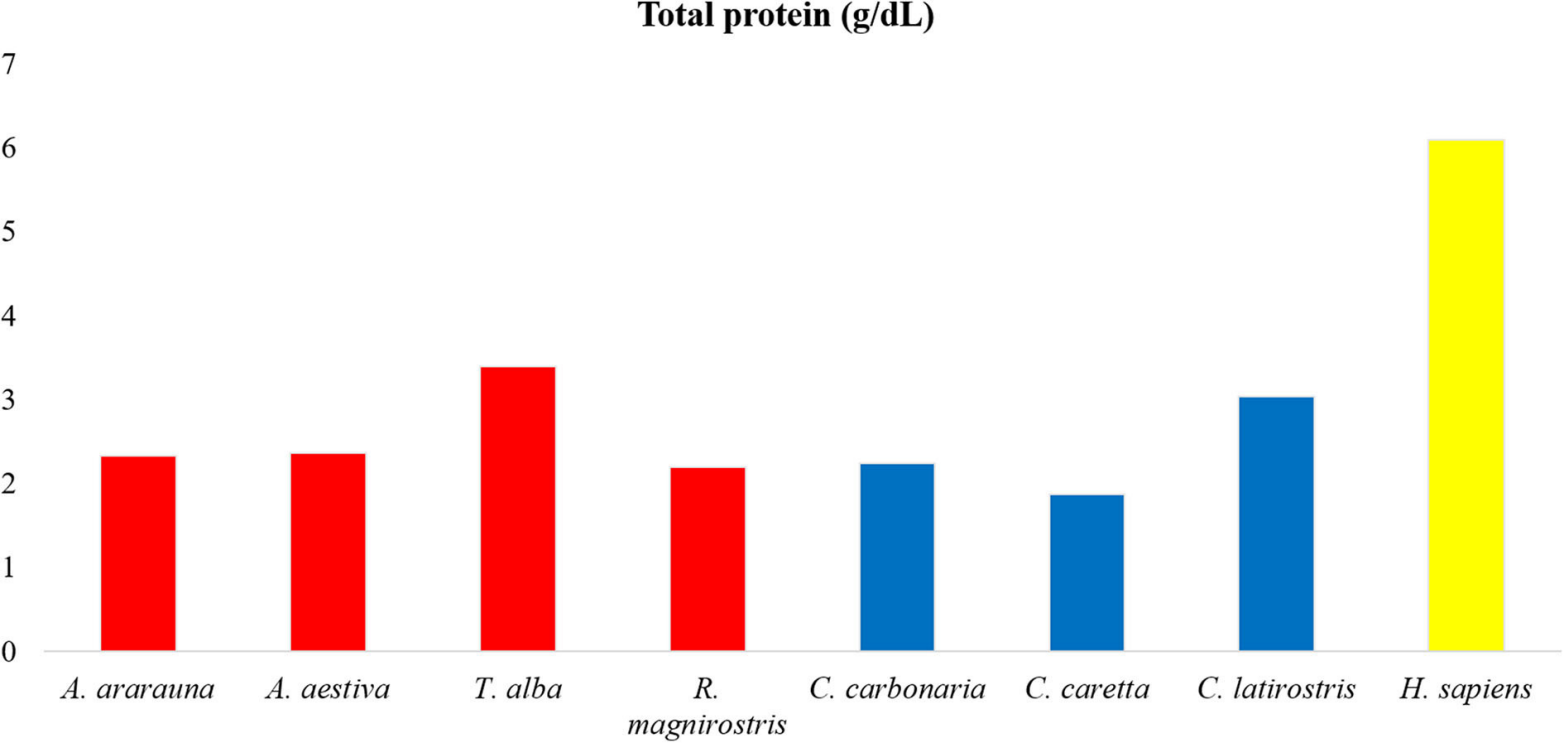
Amounts of total proteins in tears of birds, reptiles, and humans. Note high protein content in human tears, followed by *Tyto alba* and *Caiman latirostris*.

**Figure 2:**
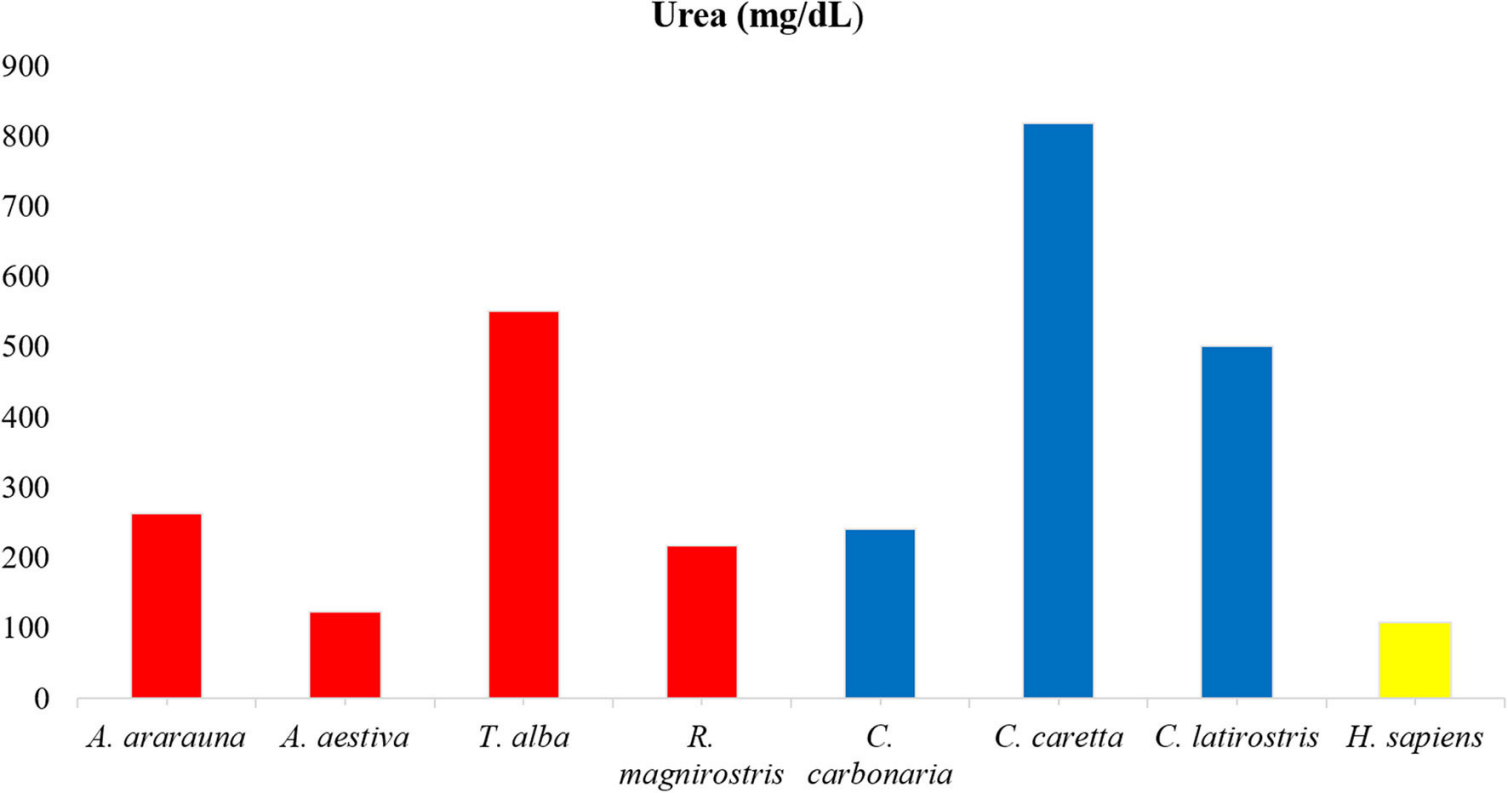
Amounts of urea in tears of birds, reptiles, and humans. Note high urea content in *Caretta caretta* tears and lower content in humans.

**Figure 3:**
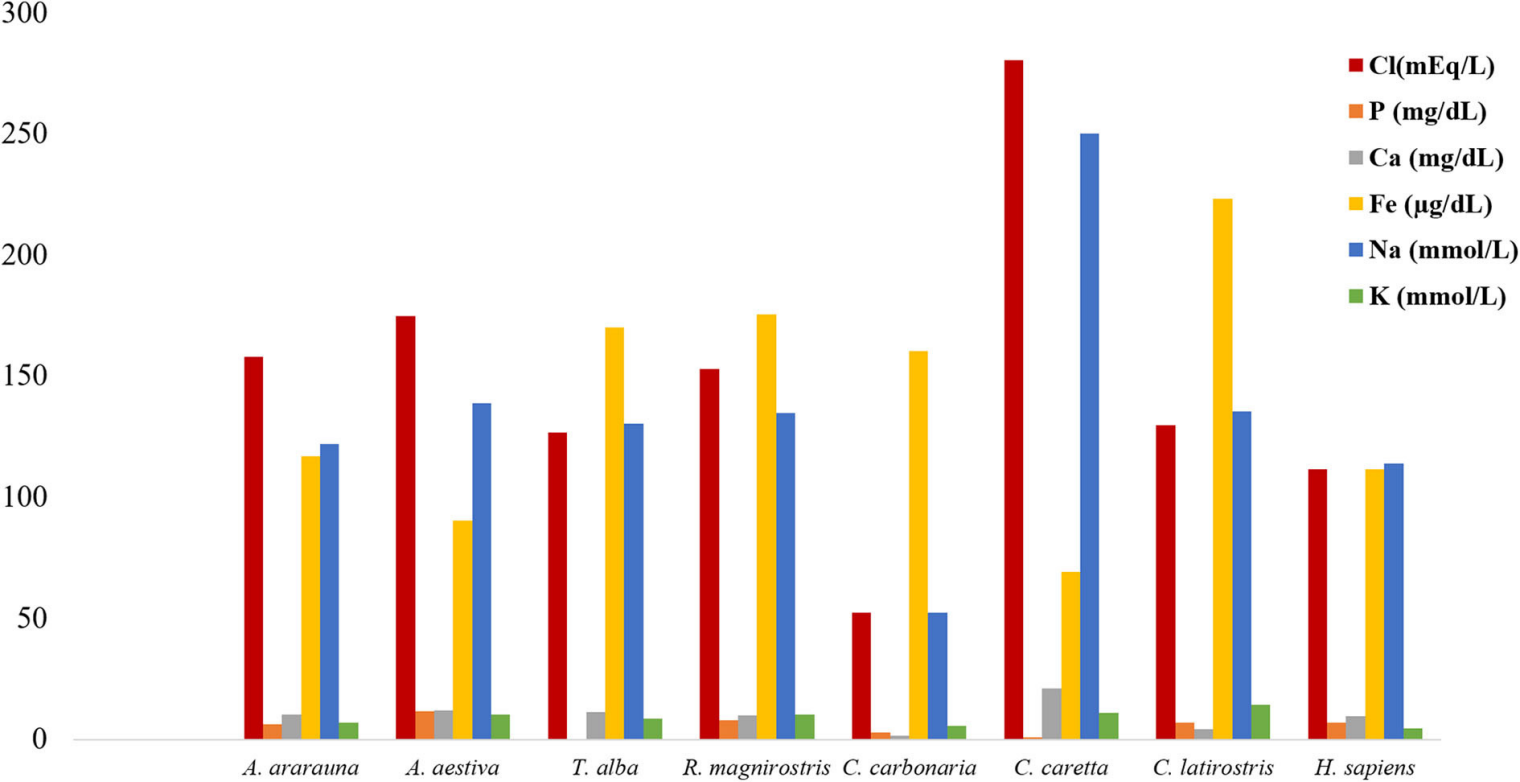
Amount of electrolytes in tears of birds, reptiles, and humans. Note increased chloride, iron, and sodium contents in tears of all species.

Dosages of total protein, urea and electrolytes were expressed as means, obtained using duplicates, and concentration values of each tear component were compared (Figures 1–3; Supplementary Table 1). Higher values of total protein were observed in human tears compared to the other species, followed by caimans and owls. Lacrimal urea was highest in sea turtles, followed by owls and caimans. The electrolyte values showed differences among species, and there were no similarities when considering phylogenetic proximity and closely related ecologic niches. However, all of the evaluated tears presented higher values of chloride, iron, and sodium compared to humans.

**Figure 4:**
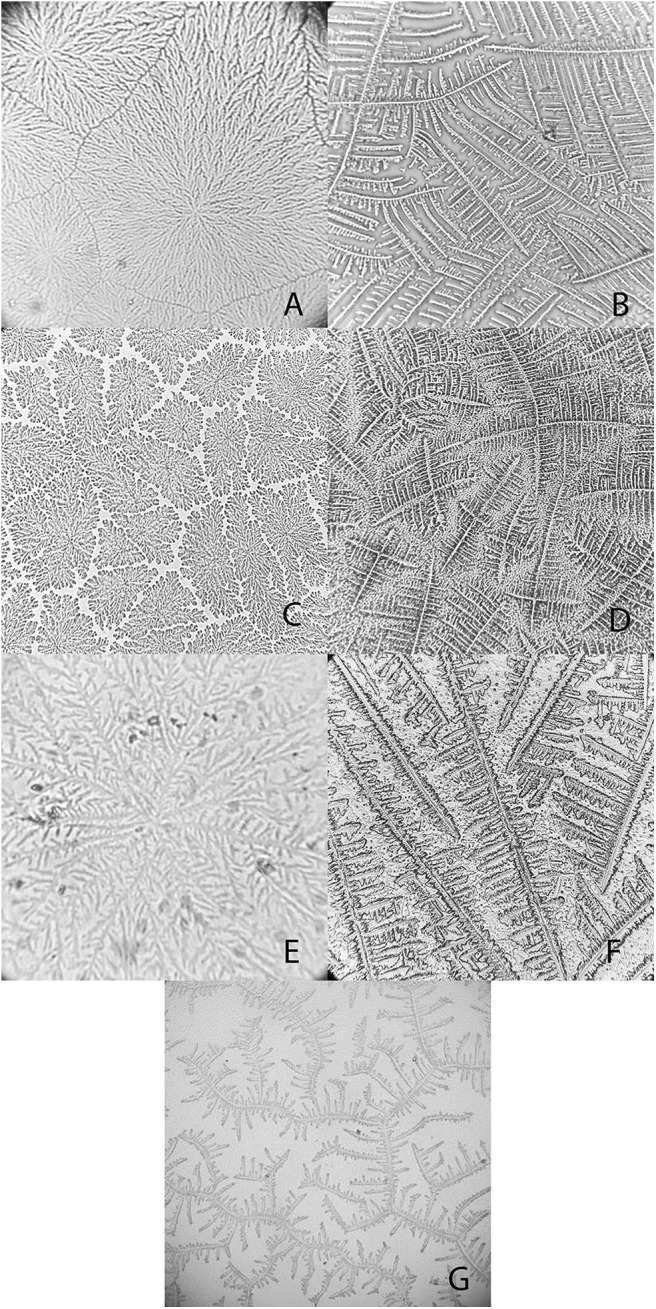
Examples of tear crystallization patterns in wild animals, graded according to Rolando and Masmali scales. **(A)** *Ara ararauna*—type I (Rolando) and grade 0 (Masmali) with uniform crystallization pattern and no spaces between branches. **(B)** *Amazona aestiva*—type II (Rolando) and grade 2 (Masmali); the main branches are coarse and the secondary branches are far from each other; there are spaces between the crystals. **(C)** *Tyto alba*—type I (Rolando) and grade 0 (Masmali); main branches are coarse and short; dense crystallization made up of branched crystals with no spaces between them. **(D)** *Rupornis magnirostris*—type II (Rolando) and grade 2 (Masmali); the main branches are more distant and less delicate; space between the crystals. **(E)** *Chelonoidis carbonaria*—type II (Rolando) and grade 1 (Masmali); the main branches are coarse and elongated; there are spaces between the secondary branches and the overlap. **(F)** *Caretta caretta*—type II (Rolando) and grade 2 (Masmali); the main branches are elongated and coarse, and the secondary branches are short; there are spaces between the crystals. **(G)** *Caiman latirostris*—type III (Rolando) and grade 3 (Masmali); the crystallization is thicker, and the crystals are more distant from each other compared to the other studied species.

Mean values for the tear crystallization time are given in Table 1. According to the Shapiro–Wilk test, the grading values were not normally distributed (*P* < 0.05); there was no difference between right and left eyes, according to the Wilcoxon test (*P* ≥ 0.083). The values of the median and interquartile range, and the frequencies for each grading scale are shown in Tables 2, 3. Aerial and terrestrial species obtained the lowest grading for Rolando and Masmali scales, however the crystallization pattern was well differentiated, highlighting *Ara ararauna* tears with a uniform crystallization and *Chelonoidis carbonaria* tears presenting large spaces between the crystals (Figure 4). Fifty per cent of the *Caiman latirostris* and 56% of the *Caretta caretta* tears were classified as grade III according to Rolando scale.

**Table 1:** Crystallization time of birds and reptiles tears (expressed in minutes).

**Species**	**Mean ± SD**
*Ara ararauna*	4.42 ± 2.00 (CI: 2.32–6.52)
*Amazona aestiva*	10.15 ± 3.40 (CI: 6.18–13.31)
*Tyto alba*	4.17 ± 3.46 (CI: 0.52–8.00)
*Rupornis magnirostris*	11.41 ± 3.12 (CI: 8.13–14.00)
*Chelonoides carbonaria*	7.15 ± 3.30 (CI: 4.11–10.20)
*Caretta caretta*	15.03 ± 7.01 (CI: 8.04–21.58)
*Caiman latirostris*	14.59 ± 5.47 (CI: 7.00–20.33)

*SD, standard deviation; CI, confidence interval*.

**Table 2:** Median and semi-interquartile range for tear ferning test (TFT) of birds and reptiles.

	**TFT**,	**TFT**,
	**Rolando scale**	**Masmali scale**
*Ara ararauna*	1.0 ± 0.0	0.0 ± 0.37
*Amazona aestiva*	2.0 ± 0.0	2.0 ± 0.0
*Tyto alba*	1.0 ± 0.37	0.5 ± 0.5
*Rupornis magnirostris*	2.0 ± 0.0	2.0 ± 0.5
*Chelonoides carbonaria*	1.5 ± 1.0	1.5 ± 0.87
*Caretta caretta*	2.5 ± 0.87	2.0 ± 1.25
*Caiman latirostris*	3.0 ± 0.87	2.0 ± 0.87

**Table 3:** Frequencies of tear ferning test grading (TFT) of birds and reptiles using Rolando and Masmali scales.

	**Rolando scale**				**Masmali scale**				
	**I**	**II**	**III**	**IV**	**0**	**1**	**2**	**3**	**4**
*Ara ararauna*	100%	–	–	–	68%	32%	–	–	–
*Amazona aestiva*	5%	90%	5%	–	–	14%	86%	–	–
*Tyto alba*	73%	20%	7%	–	33%	53%	7%	7%	–
*Rupornis magnirostris*	15%	85%	–	–	–	55%	40%	5%	–
*Chelonoides carbonaria*	40%	15%	30%	15%	10%	30%	30%	15%	15%
*Caretta caretta*	22%	22%	56%	–	22%	–	33%	45%	–
*Caiman latirostris*	5%	25%	50%	20%	5%	–	50%	25%	20%

*(–) absence of frequency*.

There were no differences in the crystallization classification between Rolando and Masmali scale according Kruskal–Wallis test (*p* ≥ 0.06). There was a moderate to substantial correlation agreement between different evaluators for Rolando scales for all animals (Table 4).

**Table 4:** Cohen's kappa agreement coefficient and *p*-value of the classification between three different examiners (A, B, and C) of birds and reptiles tear crystallization, according to the Rolando and Masmali scales.

**Species**	**Evaluator**	**Evaluator**	**Rolando scale**		**Masmali scale**	
			***k***	***p-*value**	***k***	***p-*value**
All	A	B	0.702^d^	>0.001	0.514^c^	>0.001
	A	C	0.695^d^	>0.001	0.649^d^	>0.001
	B	C	0.485^c^	>0.001	0.373^b^	>0.001
*Ara ararauna*	A	B	^*^	^–^	0.4^b^	0.114
	A	C	^*^	^–^	0.737^d^	0.16
	B	C	^*^	^–^	0.2^a^	0.490
*Amazona aestiva*	A	B	0.781^d^	>0.001	0.163^a^	0.152
	A	C	0.125^a^	0.332	0.404^c^	0.032
	B	C	0.067^a^	0.628	0.09^a^	0.482
*Tyto alba*	A	B	0.371^b^	0.104	0.845^e^	>0.001
	A	C	0.999^e^	>0.001	0.999^e^	>0.001
	B	C	0.371^b^	0.104	0.845^e^	>0.001
*Rupornis magnirostris*	A	B	0.773^d^	>0.001	0.697^d^	>0.001
	A	C	0.508^c^	0.002	0.383^b^	0.037
	B	C	0.630^d^	>0.001	0.364^b^	0.04
*Chelonoidis carbonaria*	A	B	0.507^c^	>0.001	0.03^b^	0.977
	A	C	0.452^c^	0.01	0.369^b^	0.01
	B	C	0.145^a^	0.203^b^	0.111^a^	0.270
*Caretta caretta*	A	B	0.211^b^	0.190	0.169^a^	0.184
	A	C	0.553^c^	0.032	0.675^d^	0.012
	B	C	0.593^c^	0.01	0.369^b^	0.09
*Caiman latirostris*	A	B	0.779^d^	>0.001	0.916^e^	>0.001
	A	C	0.7^d^	>0.001	0.444^c^	0.04
	B	C	0.647^d^	>0.001	0.363^b^	0.014

*Results with p < 0.05 were considered as statistically significant*.

(*) *Cohen Kappa could not be calculated because all gradings were the same among evaluators (Rolando grading 1)*.

(–) *Absence of value*.

a *Slight correlation (0.00 to 0.2)*;

b *fair correlation (0.21 to 0.4)*;

c *moderate correlation (0.41 to 0.6)*;

d *substantial correlation (0.61 to 0.8)*;

e *almost perfect correlation (0.81–1.0)*.

**Figure 5:**
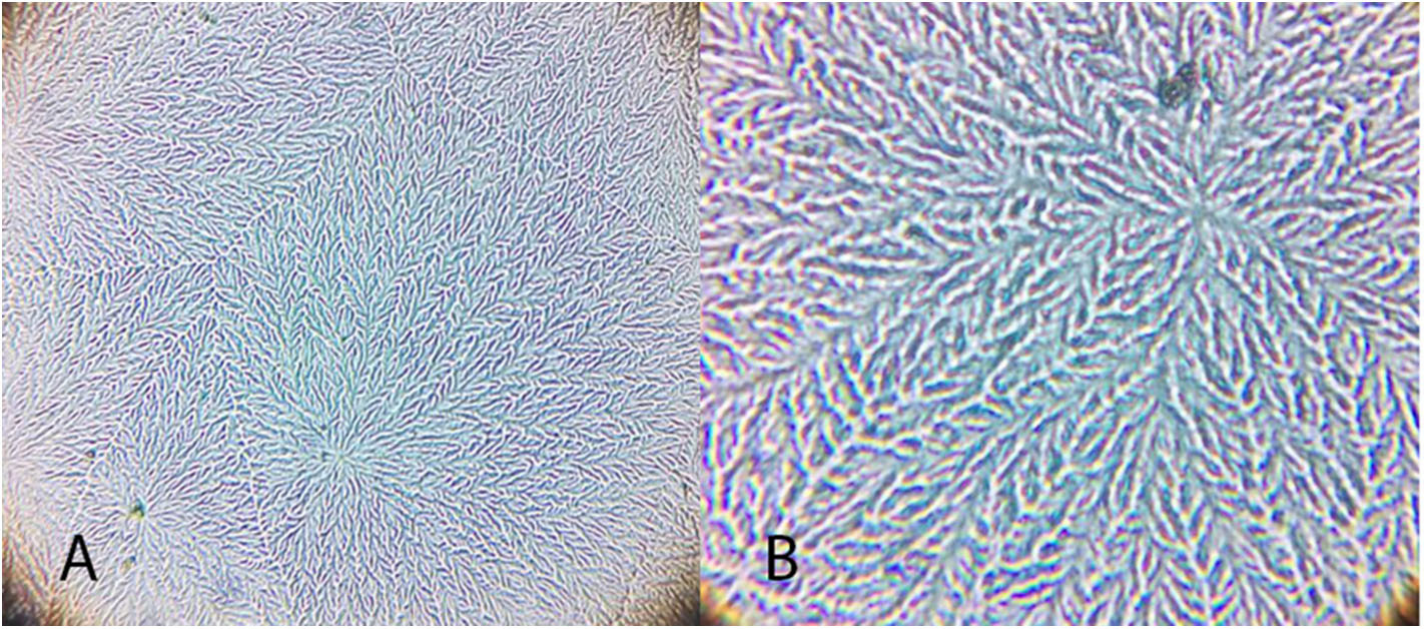
Examples of *Ara ararauna* tear ferning patterns, according to the Rolando and Masmali grading scales. **(A)** Type I (Rolando scale) and **(B)** Grading 0 (Masmali scale).

**Figure 6:**
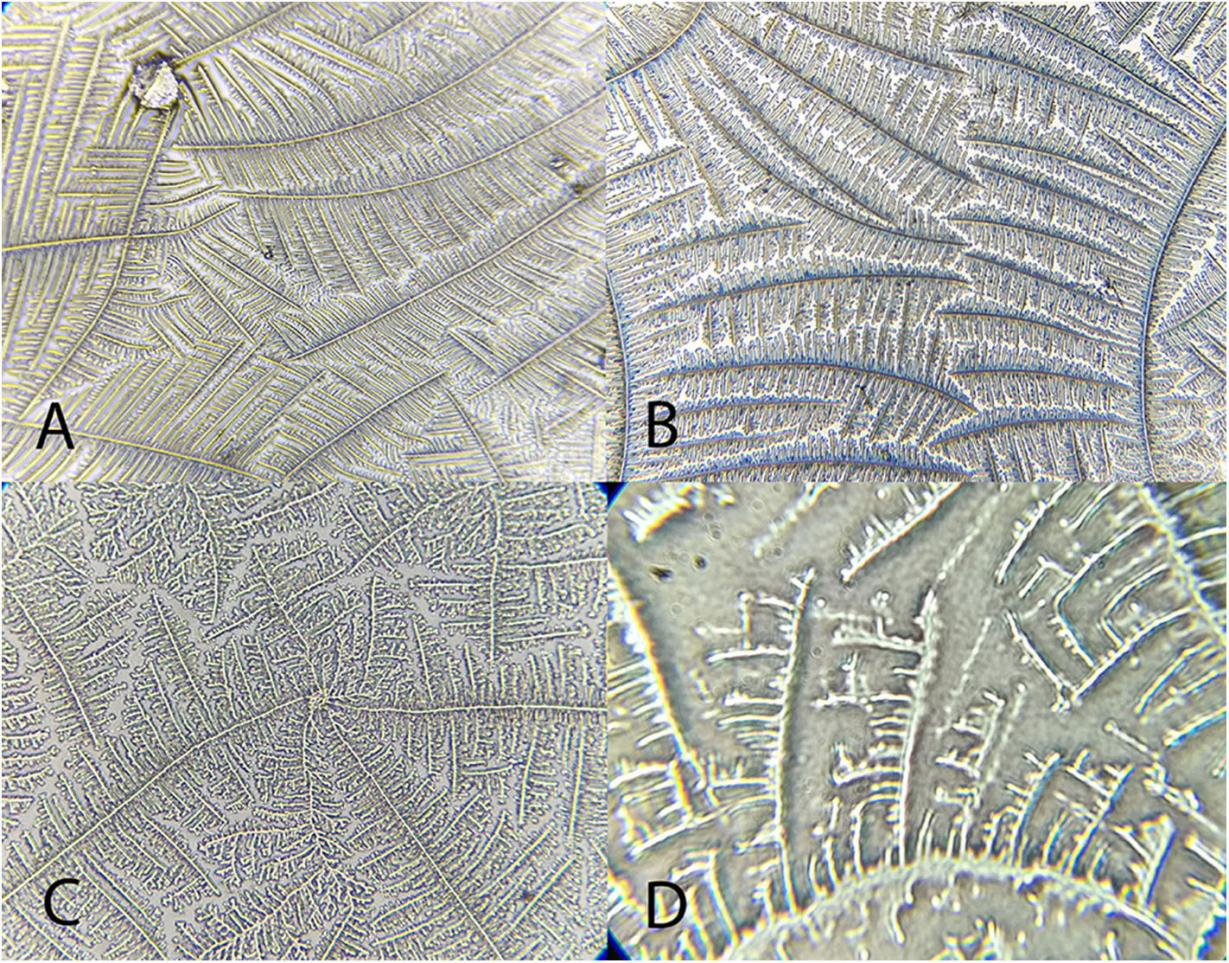
Examples of *Amazona aestiva* tear ferning patterns, according to the Rolando and Masmali grading scales. **(A)** Type I (Rolando scale); **(B)** Type II (Rolando scale), grading 2 (Masmali scale); **(C)** grading 2 (Masmali scale); **(D)** Type III (Rolando scale), grading 3 (Masmali scale).

**Figure 7:**
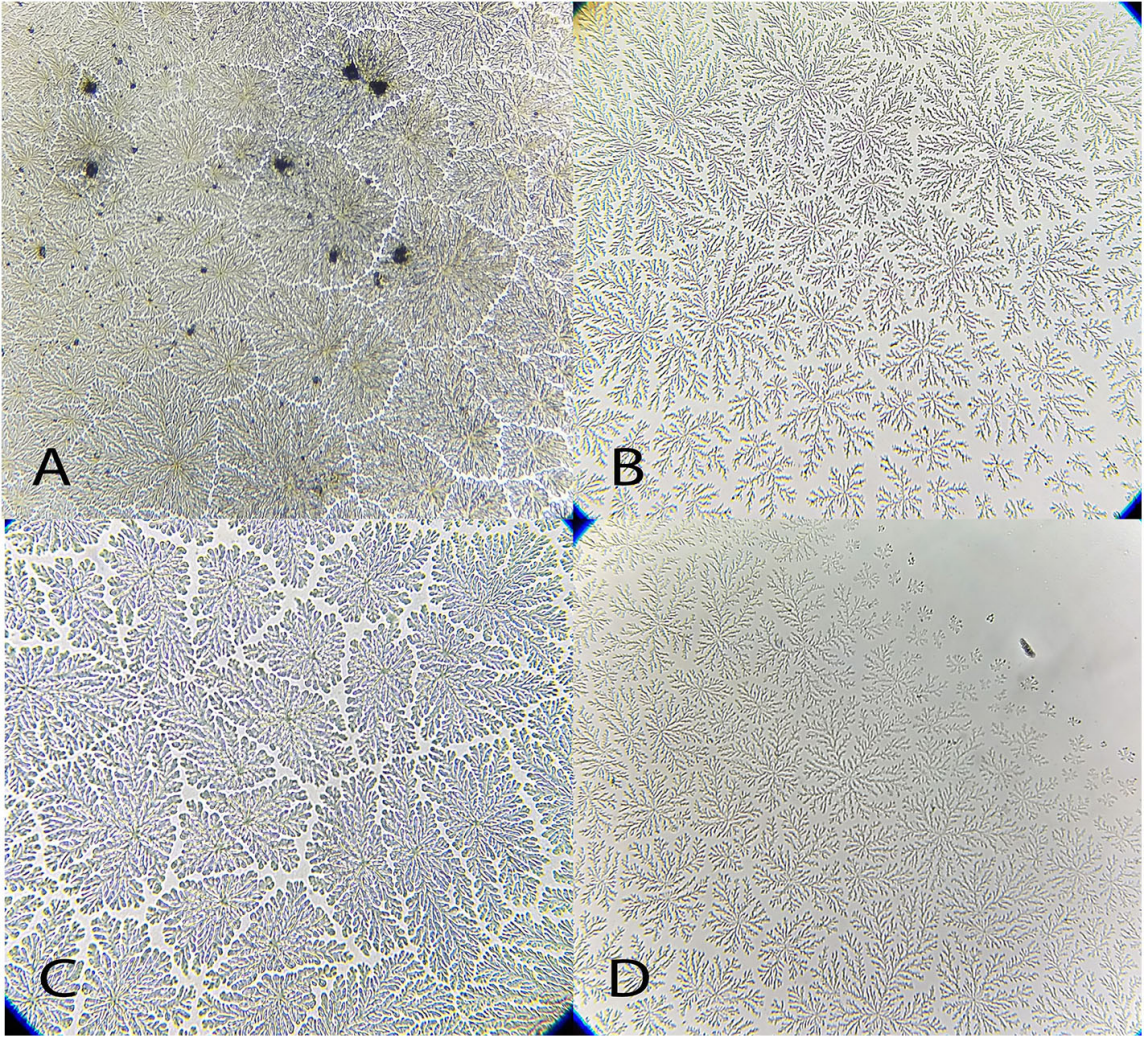
Examples of *Tyto alba* tear ferning patterns, according to the Rolando and Masmali grading scales. **(A)** Type I (Rolando scale); **(B)** Type II (Rolando scale), grading 1 (Masmali scale); **(C)** grading 1 (Masmali scale); **(D)** Type III (Rolando scale), grading 3 (Masmali scale).

**Figure 8:**
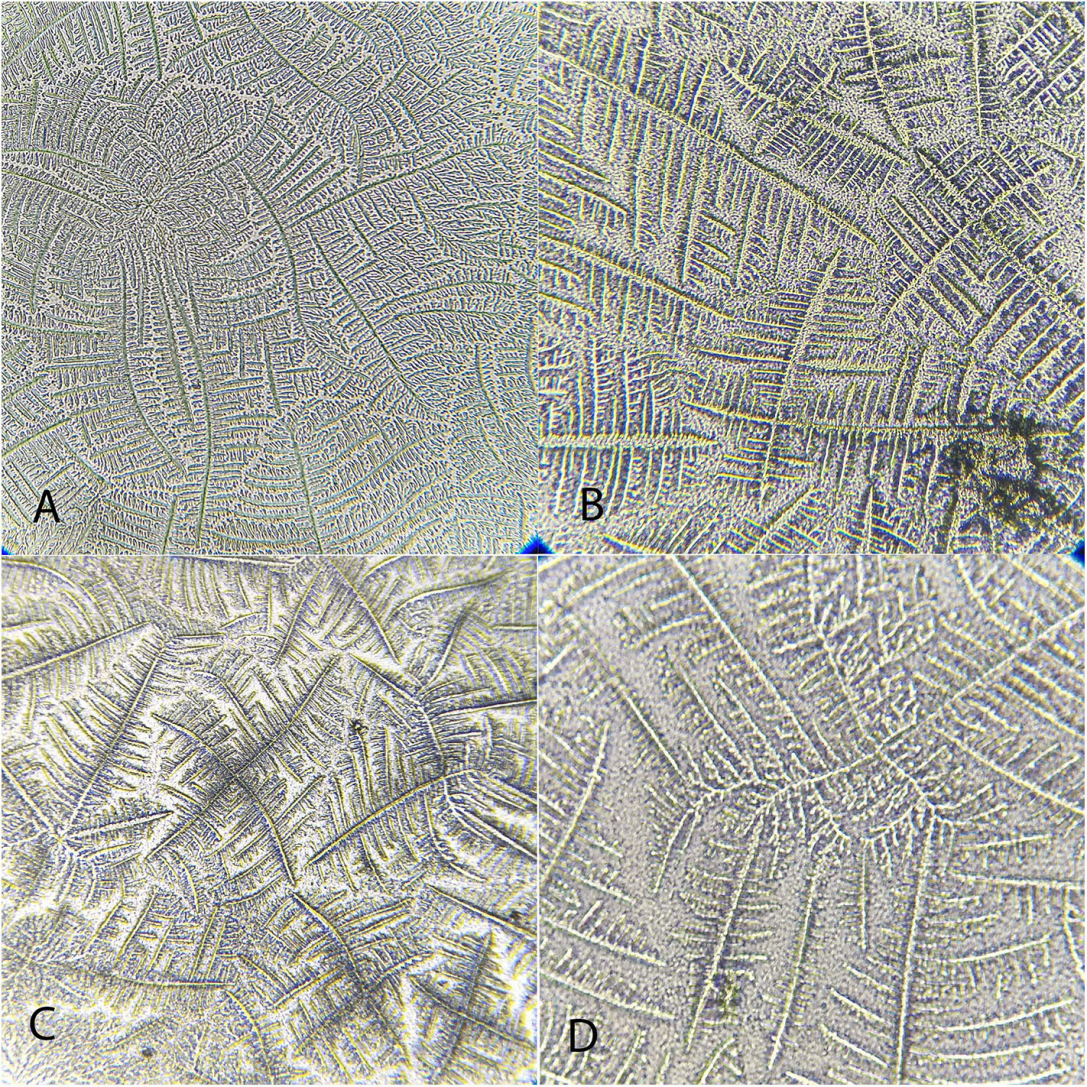
Examples of *Rupornis magnirostris* tear ferning patterns, according to the Rolando and Masmali grading scales. **(A)** Type 1 (Rolando scale), grading 1 (Masmali scale); **(B)** Grading 2 (Masmali scale); **(C)** Type II (Rolando scale); **(D)** Grading 3 (Masmali scale).

**Figure 9:**
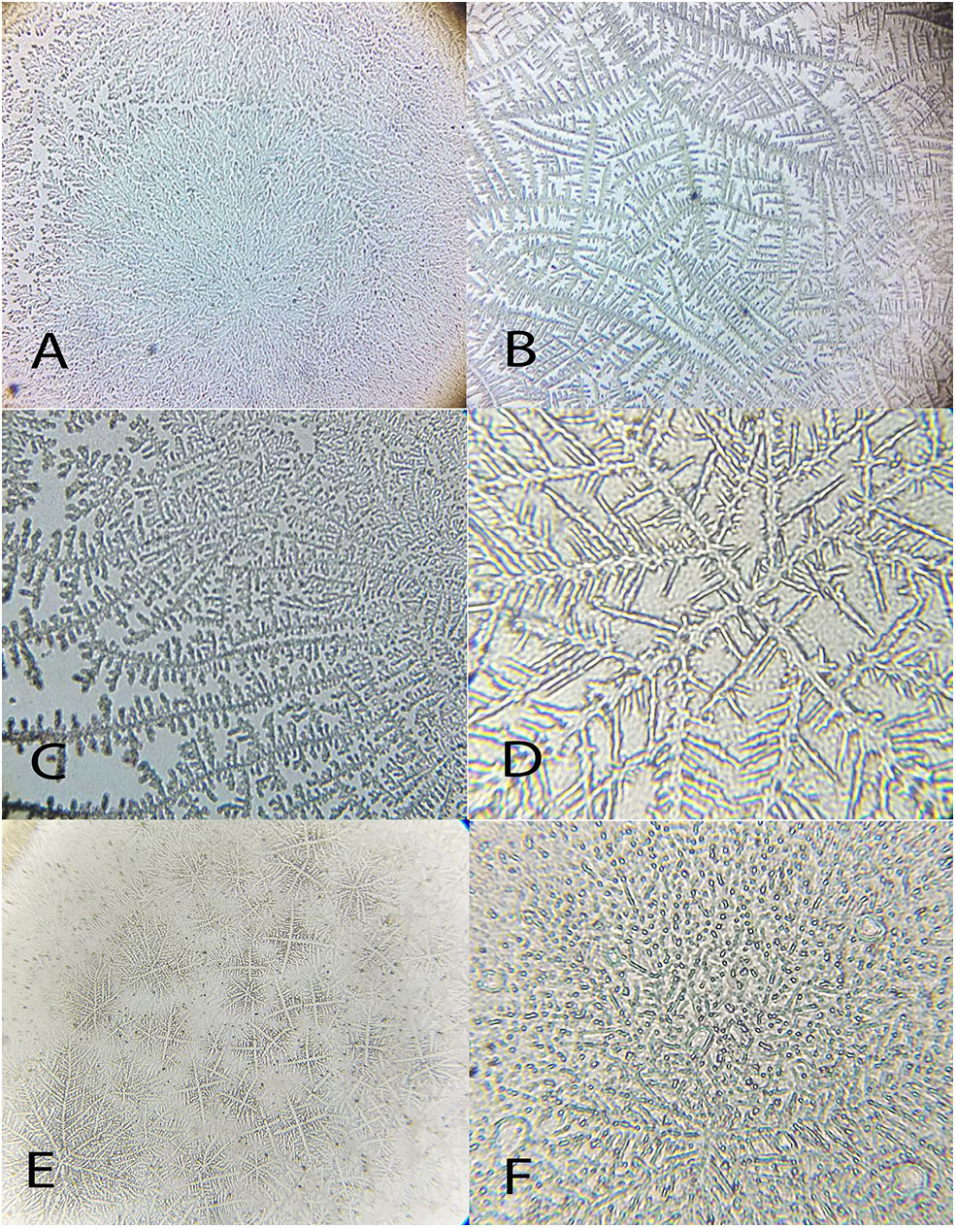
Examples of *Chelonoides carbonaria* tear ferning patterns, according to the Rolando and Masmali grading scales. **(A)** Type I (Rolando scale) grading 0 (Masmali scale); **(B)** Type II (Rolando scale), grading 1 (Masmali scale); **(C)** Type III (Rolando scale), grading 3 (Masmali scale); **(D)** Type III (Rolando scale), grading 3 (Masmali scale); **(E)** Type IV (Rolando scale), grading 3 (Masmali scale). **(F)** Grading 4 (Masmali scale).

**Figure 10:**
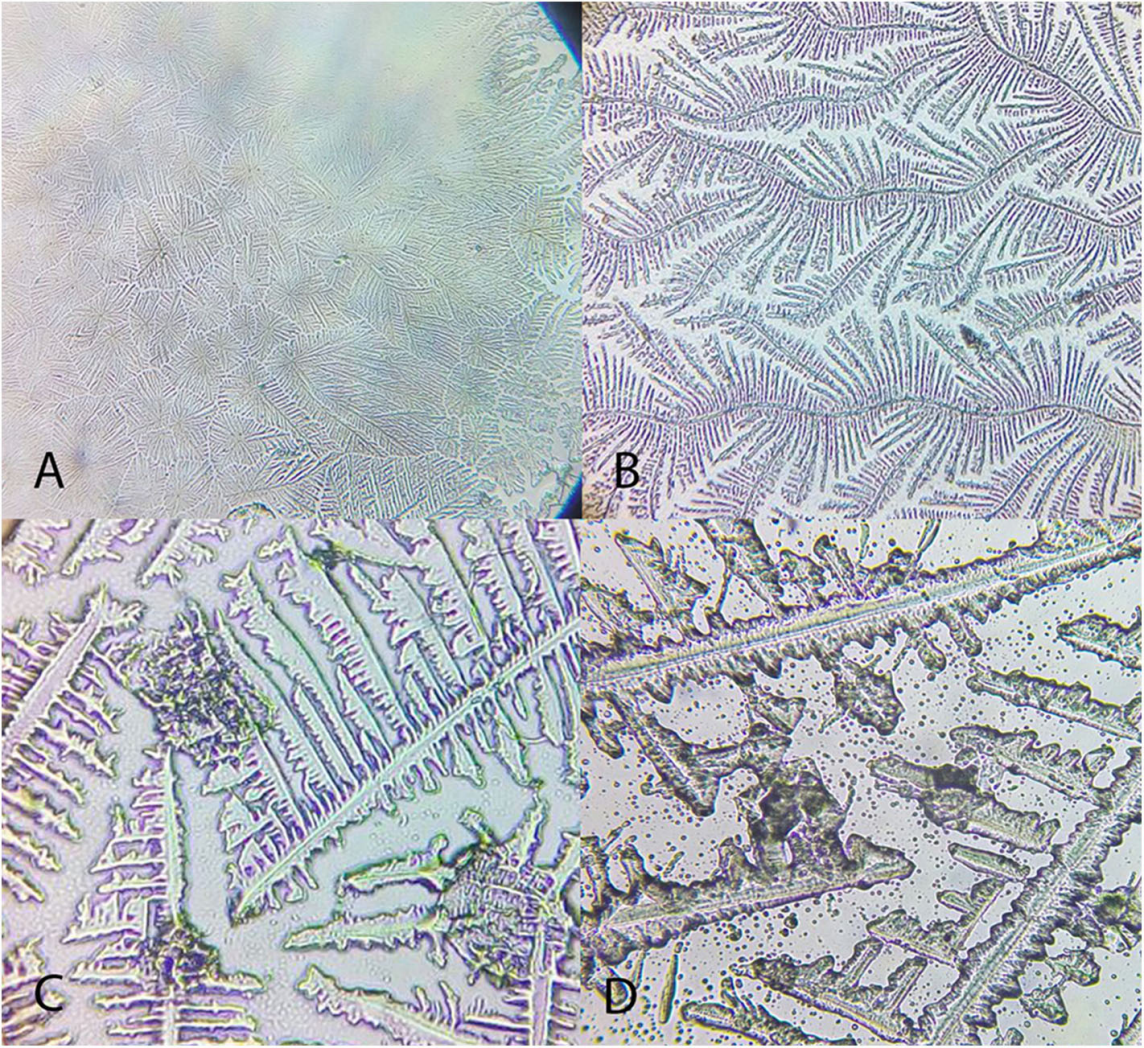
Examples of *Caretta caretta* tear ferning patterns, according to the Rolando and Masmali grading scales. **(A)** Type I (Rolando scale), grading 0 (Masmali scale); **(B)** Type II (Rolando scale), grading 1 (Masmali scale); **(C)** grading 3 (Masmali scale); **(D)** Type III (Rolando scale).

**Figure 11:**
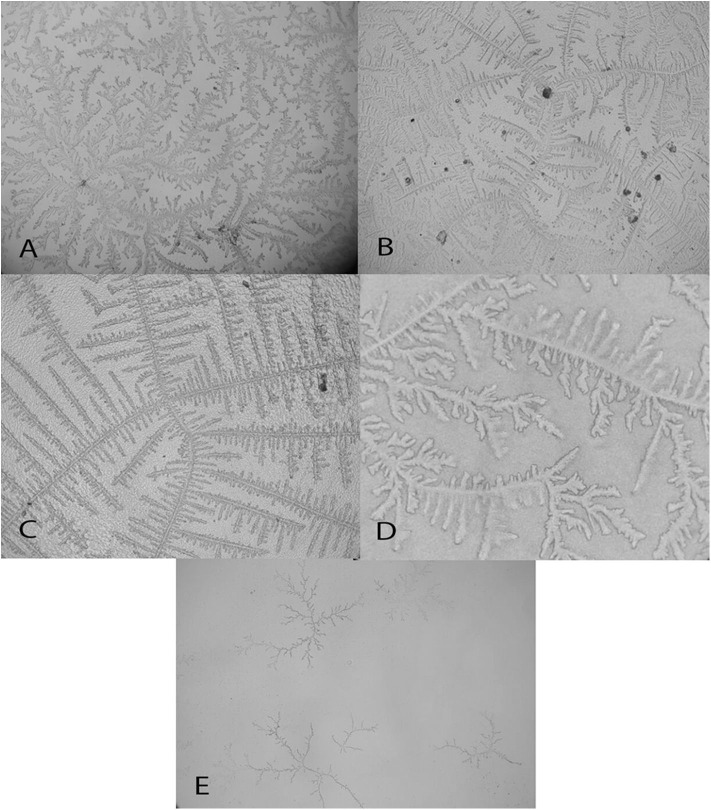
Examples of *Caiman latirostris* tear ferning patterns, according to the Rolando and Masmali grading scales. **(A)** Type II (Rolando scale), grading 1 (Masmali scale); **(B)** Type II (Rolando scale); **(C)** Type II (Rolando scale), grading 2 (Masmali scale); **(D)** Type III (Rolando scale), grading 3 (Masmali scale); **(E)** Type IV (Rolando scale), grading 4 (Masmali scale).

The following comments were made by the evaluators:

“Differences were observed between the tear crystallization patterns that received the lower grades: fine, elongated and delicate branches, and minimal space between ferns. *Ara ararauna* presented short and coarse crystals, with space between them and a crystallization pattern forming several nuclei, which were easily distinguished. The tears of *T. alba* also showed short and coarse crystals. In *R. magnirostris* and *Chelonoidis carbonaria*, the crystallization pattern was composed of coarser and less delicate crystals, with more spaces and irregularities. From the crystallization patterns with higher gradings, the *Caiman latirostris* presented bigger spaces between crystals when compared to *Caretta caretta*. The caiman also had a smaller number of crystals per evaluated field; ferns of *Caretta caretta* were coarser and there were less branches compared to *Chelonoidis carbonaria*. The crystallization pattern of *Amazona aestiva* received an intermediate grade and exhibited elongated and coarse crystals, when compared to *R. magnirostris*” (Figures 5–11).

## Discussion

The tear film is composed of biochemical subunits interacting with the ocular surface, with functions of lubrication, protection, and stability (3). The complexity of tear fluid has led to the use of qualitative methods, such as observation of the tear crystallization phenomenon, aimed at understanding the interactions between its components upon modifications in physical status. Tear comparisons can provide information on the adaptations of this fluid's composition among species, in an attempt to maintain homeostasis. Despite this being an important tool, descriptions of tear composition in wild animals are scarce. To the best of our knowledge, the results of the present study are the first to describe electrolyte composition and TFT results in birds and reptiles, compared to humans. Due to the limited number of animals in this study, the samples were pooled, and the results do not represent parameters for species.

The intrinsic contact of the tear with the environment is common to all vertebrates, however small amounts can hinder the collection process (1, 21–23). Previous studies in humans, monkeys and dogs showed similar electrophoretic profile when tears were collected through different methods collection. The choice of the collection method for each species was based in previous studies on related species (11–13, 16, 19, 23).

The tear profile of humans and other mammals is similar (13, 24), however this similarity is not observed between birds and reptiles (23). The highest concentrations of proteins were found in the tears of humans, and similar values were reported in previous studies conducted with healthy patients (around 4 to 9 g/dL) (13, 22). Among the animals, there was a heterogeneity between classes, and *Tyto alba* and *Caiman latirostris* showed the highest concentrations, which can be attributed to a widely exposed ocular surface and reduced tear production (1, 25, 26). The lowest protein concentration was seen in *Caretta caretta*, like to what was already described for sea turtles, and can be related to the contact with the marine environment (23).

Extracellular fluids, such as tears, maintain homeostasis through ionic balance and osmolarity (9, 14). In human tears, the paracellular pathway of sodium secretion is supplemented by a transcellular pathway driven by apical sodium and potassium pumps in a primary fluid-secretion model in the lacrimal gland (6), and measurements by silicon-hydrogel (SiHG) contact lens showed sodium and chloride in high concentrations, similar to what was found in this study. However, the electrolyte values reference can vary widely depending on the measurement method, such as SiHG contact lens or diurnal variation and body hydration measured through plasma and tear osmolarity (27, 28). Evaluation of the ionic concentrations in bird and reptile species showed a similar ionic balance, with increased concentrations of sodium and chloride and decreased potassium concentrations (compared to humans). Previous studies have reported differences between basal and reflex tears; however, information regarding stimuli that might induce tear production in wild animals is scarce (29). Therefore, despite the observation of ionic balance to maintain ocular surface homeostasis, additional research is needed to improve our understanding of this process.

The calcium produced in the lacrimal gland is related to intrinsic intracellular mechanisms and can be found at low concentration in tears. Among the evaluated animals' tears, calcium levels were lower than those of sodium and potassium, which were at similar concentrations, the latter two exhibiting important intracellular function in humans (6). Studies related to lacrimal phosphorus are scarce and limited to reports of low concentrations of phosphate anions (30). Here, low phosphate concentrations were observed in the tears of all studied species, and among the evaluated electrolytes, iron had higher concentration values when compared to the sodium and potassium ones. This shows the importance of iron, which is correlated to proteins in mammalian tears, such as lactoferrins (3, 4). However, further studies of iron's function in the ionic balance are needed.

Masmali et al. (2) affirmed that crystallization is derived from the electrolytes that make up the tear fluid, and from the macromolecule and protein migration to the crystal edges in humans. When this evaluation method was applied to patients with ocular surface diseases, hyperosmolarity was suggested to produce higher grades in those individuals (9, 10). Despite the differences in electrolyte concentrations in humans vs. other the studied animals, there were similarities in ionic balance, so the other components may hold similar importance for the crystallization phenomenon. However, differences between arrangements and the electrolyte values revealed that the crystal pattern might result from the interaction of these molecules.

Other tear components, including proteins and nitrogenated products, such as urea, can interact with electrolytes (7). Tears possess a heterogeneous protein composition and there is meant to be unrestricted passage of urea through the lacrimal gland (31). As these elements may interfere with crystal formation, the observed differences between grading, arrangements, and evaluator impressions reflect the complexity of the tear film composition, because exogenous variables, such as temperature and humidity, are strictly monitored during the TFT (2, 32).

Crystallization occurs when a tear is placed on a glass slide and the solvent is evaporated. Several variables, including temperature and humidity, can interfere with this time-dependent measurement (32). However, the crystallization could not be proportionally correlated to the obtained classifications, and crystallization time probably results from the microcomponents in the tears. The crystallization process requires a slow crystal-formation rate, low solution viscosity and low levels of impurity to allow diffusion of solutes. If these conditions are not met, growth of dendritic crystals may occur (2).

There is no consensus in the literature regarding the ideal crystallization time, and variations from 7 to 10 min, described for humans and monkeys, respectively, have been reported (13). The time range for horses, dogs and cats tear crystallization were around 10 to 18 min (12, 18, 33), and all aerial and terrestrial species presented a similar pattern. The crystallization time of the *Caiman latirostris* and *Caretta caretta* (a semi-aquatic and a marine species, respectively) tears was greater than the previously reported species, and possible differences in the composition of these tears may have delayed the tear drying process.

The sea turtle and caiman crystallization arrangements were thicker than those of the aerial and terrestrial species. This suggests that animals in contact with the aquatic environment have a specific tear composition aimed at maintenance and stability, including, for example, mucus. In human tears, thicker crystals result mainly from changes induced by the increased presence of mucus or macromolecules (1, 11, 14, 32).

In this study, a type III crystallization pattern (Rolando scale) was observed for healthy *Caiman latirostris*, the same as for *Amazona aestiva* and *R. magnirostris*. Raposo et al. (13) gave healthy capuchin monkeys tears a grade of III, attributed to normal species-specific particularities, and suggested an increment of 0.1 points between degrees and classification times, as proposed for humans, to increase the test's sensitivity while decreasing its subjectivity (9).

Although most of the results were similar among animals and humans, with grades of I (Rolando scale) and 0 and 1 (Masmali scale)—including the crystals obtained for *Ara ararauna* and *T. alba* with similar grades—the arrangements showed distinct conformations. Tear composition can also be affected by food intake (34). Therefore, when comparing the crystals obtained from the tears of *T. alba* and *R. magnirostris* (both carnivorous predatory birds) differences in classification and pattern were observed between then. From our observations of the wild species vs. human tears, the primary and secondary branches and the transition areas, which are the evaluation criteria proposed by Rolando (10) and Masmali et al. (2), do not seem to apply to tear crystallization patterns for healthy birds and reptiles.

The use of two numeric grading systems have improved the evaluation of tear ferning patterns, but the assumptions made by each evaluator have a direct relationship with their ability to identify or detect changes in the crystallization process. It has been shown in this study that there was an inter-observer substantial to moderate correlation agreement, and this situation led to the conclusion that the classifications of the ferning patterns for both scales can be significantly reproducible.

There were variations between the scales, and they are derived from the similarity between what is considered to classify the TFT, such as spacing, thickness, number of cores, and length of branches. Since some species did not show differences for a same characteristic, the evaluators experienced some difficulties in the use of scales for each species. In general, in species with tear crystallization characteristics or arrangement similar to those of humans, the Rolando scale was more uniform among the evaluators, and there was a greater difference for the results obtained with the use of the Masmali scale.

In conclusion, the ionic balance of the tear fluid of birds and reptiles is similar to that described for humans except for higher sodium and chloride contents. However, the tear crystals showed differences in arrangement between the wild species and humans. The obtained classifications revealed that major degrees and types are not necessarily related to the lacrimal disease picture in wild animals. The results showed differences among bird and reptile tears, and this information should be supported by further studies on wild animal tears.

## Data Availability Statement

The raw data supporting the conclusions of this manuscript will be made available by the authors, without undue reservation, to any qualified researcher.

## Ethics Statement

The studies involving human participants were reviewed and approved by Ethics Committee in Research of the Institute of Health Science, Federal University of Bahia. The patients/participants provided their written informed consent to participate in this study. The animal study was reviewed and approved by Ethics Committee on Animal Experimentation of the School of Veterinary Medicine and Zootechnology of UFBA.

## Author Contributions

AO and RP conceived and designed the study. AL, AR, NA, and MM carried out investigation and methodology. The data validation was made by AO, RP, and AM. All authors have read and accepted the manuscript as it is presented to the journal.

## Conflict of Interest

The authors declare that the research was conducted in the absence of any commercial or financial relationships that could be construed as a potential conflict of interest.
