# Supplementary material for: Comparison of Electrolyte Composition and Crystallization Patterns in Bird and Reptile Tears
Source: Front Vet Sci. 2020 Aug 13;7:574. doi: 10.3389/fvets.2020.00574 (PMC7438592; doi:10.3389/fvets.2020.00574)
Supplement: Supplementary file 1 [file Table_1.docx]

**TABLE 1** Dosage of total proteins, electrolytes and urea of tears of birds, reptiles and humans.

*Test not performed due to sample shortage.

| Species | Total Proteins  (g/dL) | Chloride  (mEq/L) | Phosphor (mg/dL) | Calcium (mg/dL) | Iron  (µg/dL) | Sodium (mmol/L) | Potassium  (mmol/L) | Urea (mg/dL) |  |
| --- | --- | --- | --- | --- | --- | --- | --- | --- | --- |
| *Ara ararauna* | 2.33 | 158.05 | 6.58 | 10.31 | 117.12 | 122.07 | 7.14 | 262.72 |  |
| *Amazona aestiva* | 2.36 | 174.98 | 11.96 | 12.09 | 90.44 | 138.94 | 10.42 | 123.80 |  |
| *Tyto alba* | 3.39 | 126.99 | -* | 11.31 | 170.24 | 130.50 | 8.74 | 551.21 |  |
| *Rupornis magnirostris* | 2.19 | 152.99 | 8.09 | 9.99 | 175.56 | 135.00 | 10.47 | 217.27 |  |
| *Chelonoides carbonaria* | 2.24 | 52.46 | 3.18 | 1.80 | 160.37 | 52.35 | 5.71 | 240.30 |  |
| *Caretta caretta* | 1.87 | 280.73 | 1.20 | 21.26 | 69.16 | 250.26 | 11.07 | 817.57 |  |
| *Caiman latirostris* | 3.04 | 130.04 | 7.23 | 4.26 | 223.44 | 135.56 | 14.37 | 500.60 |  |
| *Homo sapiens* | 6.1 | 111.82 | 7.19 | 9.81 | 111.72 | 114.20 | 4.89 | 107.27 |  |
